# Supplementary material for: Nonlocal effects in plasmon-emitter interactions
Source: Nanophotonics. 2024 Apr 15;13(15):2741–51. doi: 10.1515/nanoph-2023-0575 (PMC11501547; doi:10.1515/nanoph-2023-0575)
Supplement: Supplementary file 1 — Supplementary Material Details [file j_nanoph-2023-0575_suppl_001.pdf]

Nonlocal effects in plasmon-emitter interactions

– SUPPORTING INFORMATION –

Mikkel Have Eriksen,[1] Christos Tserkezis,[1] N. Asger Mortensen,[1,2] and Joel D. Cox[1,2,*]

[1] POLIMA—Center for Polariton-driven Light–Matter Interactions, University of Southern Denmark, Campusvej 55, DK-5230 Odense M, Denmark

[2] Danish Institute for Advanced Study, University of Southern Denmark, Campusvej 55, DK-5230 Odense M, Denmark

(Dated: March 13, 2024)

We elaborate on the macroscopic quantum electrodynamic formalism used to describe the Purcell factor and Lamb shift in terms of the reflected part of the classical dyadic Green's tensor, for which we provide additional theoretical details related to its dependence on quantum surface surface effects captured by Feibelman d-parameters in fully retarded and quasistatic descriptions of both planar and spherical geometries. We also show a comparison of the Purcell factors predicted in the quasistatic regime using the analytical d-parameters based on specular reflection and hydrodynamic models with those obtained from direct electrodynamic calculations applying the same level of theory.

CONTENTS

S1. Macroscopic quantum electrodynamics – Purcell factor and Lamb shift 1

S2. Optical response of planar geometries 3

S3. Optical response of spherical geometries 4
- A. Quasistatic regime 5
  - 1. Homogeneous sphere 5
  - 2. Core-shell nanoparticle 5
- B. Mie Theory 6
  - 1. Homogeneous sphere 7
  - 2. Core-shell nanoparticle 8

S4. Validity of the quasistatic approximation 9

S5. Comparison of surface response corrections to hydrodynamic simulations 9

References 11

S1. MACROSCOPIC QUANTUM ELECTRODYNAMICS – PURCELL FACTOR AND LAMB SHIFT

The Hamiltonian describing a two-level system (TLS) coupled to an arbitrary photonic environment is given by [1–4]

$$\mathcal{H} = \hbar \sum_{j=1}^{2} \varepsilon_j \left|j\right\rangle \left\langle j\right| + \hbar \int_0^\infty d\omega\, \omega \int d^3\mathbf{r}\, \hat{\mathbf{f}}_\omega^\dagger(\mathbf{r}) \cdot \hat{\mathbf{f}}_\omega(\mathbf{r}) - \hbar \int_0^\infty d\omega\, \hat{\mathbf{p}} \cdot \left[\hat{\mathbf{E}}_\mathrm{R}(\mathbf{r},\omega) + \hat{\mathbf{E}}_\mathrm{R}^\dagger(\mathbf{r},\omega)\right]. \tag{S1}$$

In the above expression, the first term describes the bare TLS characterized by stationary states $\left|j\right\rangle$ with energies $\hbar\varepsilon_j$ for $j \in \{1,2\}$; the second term is the reservoir Hamiltonian, expressed in terms of bosonic field operators $\hat{\mathbf{f}}_\omega^\dagger(\mathbf{r})$ and $\hat{\mathbf{f}}_\omega(\mathbf{r})$ that describe the creation and annihilation, respectively, of an electrodynamic vacuum excitation in the presence of an absorptive and dispersive medium; the remaining term accounts for the interaction between the reservoir and the TLS in the dipole approximation, which is quantified by the projection of the TLS dipole operator $\hat{\mathbf{p}}$ on the quantized radiation field operator

$$\hat{\mathbf{E}}_\mathrm{R}(\mathbf{r},\omega) = \mathrm{i}\sqrt{\frac{\hbar}{\pi\epsilon_0}}\frac{\omega^2}{c^2} \int d^3\mathbf{r}' \sqrt{\mathrm{Im}\{\chi_\omega(\mathbf{r}')\}}\mathcal{G}_\omega(\mathbf{r},\mathbf{r}') \cdot \hat{\mathbf{f}}_\omega(\mathbf{r}'), \tag{S2}$$

* Joel D. Cox: cox@mci.sdu.dk

where χ_ω is the susceptibility of the environment and $\mathcal{G}_\omega$ is the classical dyadic Green's tensor [5]. The Green's tensor describes the electromagnetic response at a position $\mathbf{r}$ due to an excitation at $\mathbf{r}'$, and is found for a given photonic environment as the solution to the homogeneous wave equation

$$\nabla \times \frac{1}{\mu(\mathbf{r},\omega)} \nabla \times \mathcal{G}_\omega(\mathbf{r},\mathbf{r}') - \frac{\omega^2}{c^2}\epsilon(\mathbf{r},\omega)\mathcal{G}_\omega(\mathbf{r},\mathbf{r}') = \mathbb{1}\delta(\mathbf{r}-\mathbf{r}'), \tag{S3}$$

where $\mu(\mathbf{r},\omega)$ and $\epsilon(\mathbf{r},\omega)$ are the relative permeability and permittivity of the environment, respectively. Here we consider exclusively nonmagnetic media, and so the relative permeability is taken as unity.

A master equation for the density matrix ρ in Lindblad form is found by tracing over the reservoir degrees of freedom, invoking the Born–Markov approximation, assuming weak excitation (i.e., at most one quantum in the system), and moving to an interaction picture:

$$\frac{\partial \hat{\rho}}{\partial t} = \frac{\Gamma}{2}\left(2\left|1\right\rangle\left\langle 2\right|\hat{\rho}\left|2\right\rangle\left\langle 1\right| - \left|2\right\rangle\left\langle 2\right|\hat{\rho} - \hat{\rho}\left|2\right\rangle\left\langle 2\right|\right) - \mathrm{i}\delta\varepsilon[\left|2\right\rangle\left\langle 2\right|, \hat{\rho}], \tag{S4}$$

where the total spontaneous emission rate

$$\Gamma = \Gamma_0 + \frac{2\mu_0}{\hbar}\varepsilon^2 \mathrm{Im}\big\{\mathbf{p}^* \cdot \mathcal{G}_\varepsilon^{\mathrm{ref}}(\mathbf{r},\mathbf{r}) \cdot \mathbf{p}\big\} \tag{S5}$$

is expressed in terms of the vacuum decay rate $\Gamma_0 = \varepsilon^3|\mathbf{p}|^2/3\pi\epsilon_0\hbar c^3$, defined by the emitter transition frequency $\varepsilon \equiv \varepsilon_2 - \varepsilon_1$, the transition dipole moment $\mathbf{p} \equiv \langle 1|\hat{\mathbf{p}}|2\rangle = \langle 2|\hat{\mathbf{p}}|1\rangle$, and the reflected part of the total classical Green's tensor $\mathcal{G}_\varepsilon = \mathcal{G}_\varepsilon^0 + \mathcal{G}_\varepsilon^{\mathrm{ref}}$, the latter contributing to the Purcell factor Γ/Γ_0 that quantifies the change in the TLS decay rate due to the nanophotonic environment [6], while the shift in the bare transition frequency due to the photonic environment—the Lamb shift—is

$$\delta\varepsilon = \frac{\mu_0}{\pi\hbar}\mathcal{P}\int_0^\infty d\omega \frac{\omega^2}{\varepsilon - \omega}\mathrm{Im}\big\{\mathbf{p}^* \cdot \mathcal{G}_\omega^{\mathrm{ref}}(\mathbf{r},\mathbf{r}) \cdot \mathbf{p}\big\}, \tag{S6}$$

where $\mathcal{P}$ denotes the principal value of the integral. In Eq. (S6), we have neglected the contribution of the divergent homogeneous Green's tensor $\mathcal{G}_\varepsilon^0$ by absorbing it into the definition of the transition frequency, as its proper treatment would otherwise require mass-renormalization techniques, while the vacuum Lamb shift is expected to be negligible compared to the photonic Lamb shift [7].

Properly taking into account the negative frequency terms in the integral for the Lamb shift, one finds that Eq. (S6) may be rewritten as [8]

$$\delta\varepsilon = -\frac{\mu_0}{\hbar}\varepsilon^2 \mathrm{Re}\big\{\mathbf{p}^* \cdot \mathcal{G}_\varepsilon^{\mathrm{ref}}(\mathbf{r},\mathbf{r}) \cdot \mathbf{p}\big\} - \frac{\mu_0}{\pi\hbar}\int_0^\infty d\kappa \frac{\varepsilon\kappa^2}{\kappa^2 + \varepsilon^2}\mathrm{Re}\big\{\mathbf{p}^* \cdot \mathcal{G}_{\mathrm{i}\kappa}^{\mathrm{ref}}(\mathbf{r},\mathbf{r}) \cdot \mathbf{p}\big\}. \tag{S7}$$

Assuming that the primary contribution to the original principal value integral originates near the transition frequency, the integral in the above expression can be neglected and the Lamb shift reduces to

$$\delta\varepsilon \approx -\frac{\mu_0}{\hbar}\varepsilon^2 \mathrm{Re}\big\{\mathbf{p}^* \cdot \mathcal{G}_\varepsilon^{\mathrm{ref}}(\mathbf{r},\mathbf{r}) \cdot \mathbf{p}\big\}. \tag{S8}$$

In Fig. S1 we compare the Lamb shift predicted by Eqs. (S7) and (S8) for a TLS in vacuum a distance h above a single gold interface with a normally-oriented transition dipole moment. The results indicate that the integral in Eq. (S7) contributes primarily with a constant offset that can be absorbed into the emitter transition frequency, and so we conclude that the approximation of Eq. (S8) is well-justified for the plasmonic systems we consider here.

As reported in Refs. [7, 10], the spontaneous emission spectrum produced by an initially excited TLS located at a position $\mathbf{r}$ can be expressed analytically as

$$S(\mathbf{r}_\mathrm{D},\omega) = \left|\frac{\mu_0\omega^2\mathbf{p}^* \cdot \mathcal{G}_\omega(\mathbf{r}_\mathrm{D},\mathbf{r})(\omega+\varepsilon)}{\varepsilon^2 - \omega^2 - \mathrm{i}\omega\gamma_0 - 2\mu_0\varepsilon\omega^2\mathbf{p}^* \cdot \mathcal{G}_\omega(\mathbf{r},\mathbf{r}) \cdot \mathbf{p}/\hbar}\right|^2, \tag{S9}$$

where $\mathbf{r}_\mathrm{D}$ is the position at which the signal is detected and γ_0 is the non-radiative broadening of the TLS. In particular, the above expression is obtained using the Wiener–Khinchin theorem in the weak excitation approximation [11], and can capture non-Markovian effects associated with, e.g., the propagation of light from the TLS to a detector.

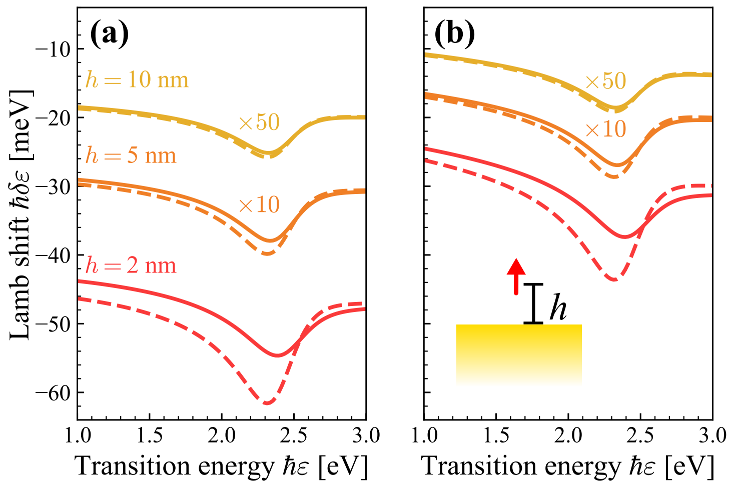

FIG. S1. **Full and simplified calculations of the Lamb shift.** We contrast the Lamb shift calculated using (a) the full expression of Eq. (S7) and (b) the approximation of Eq. (S8) for a dipole in air positioned a distance h above a gold half-space using the retarded (solid curves) and quasistatic (dashed curves) Green's functions in Eqs. (S11a) and (S16a), respectively. The dipole moment is taken as $1\,e{\cdot}$nm and the permittivity of gold is given by the model reported in Ref. [9].

S2. OPTICAL RESPONSE OF PLANAR GEOMETRIES

The quantum electrodynamic behavior of a quantum emitter (QE) located a distance $z > 0$ above stratified media with translational symmetry in the x-y plane and uppermost interface at $z = 0$ is characterized by the projection of the transition dipole moment on the reflected part of the Green's dyadic at the dipole location, which can be decomposed according to

$$\mathbf{p}^* \cdot \mathcal{G}^{\rm ref}_{\omega} \cdot \mathbf{p} = \mathcal{G}^{\rm ref}_{\omega,\parallel} |p_\parallel|^2 + \mathcal{G}^{\rm ref}_{\omega,\perp} |p_\perp|^2, \tag{S10}$$

where the subscripts $\{\parallel, \perp\}$ indicate vector components that are parallel and perpendicular to the interface and

$$\mathcal{G}^{\rm ref}_{\omega,\perp} = \frac{\rm i}{4\pi k_1^2} \int_0^\infty dk_\parallel \frac{k_\parallel}{k_{1,\perp}} k_\parallel^2 r_{\rm p}(k_\parallel) {\rm e}^{{\rm i}2k_{1,\perp}z} \tag{S11a}$$

$$\mathcal{G}^{\rm ref}_{\omega,\parallel} = \frac{\rm i}{8\pi k_1^2} \int_0^\infty dk_\parallel \frac{k_\parallel}{k_{1,\perp}} \left[k_1^2 r_{\rm s}(k_\parallel) - k_{1,\perp}^2 r_{\rm p}(k_\parallel)\right] {\rm e}^{{\rm i}2k_{1,\perp}z} \tag{S11b}$$

are the associated Green's functions, expanded in parallel components $k_\parallel = \sqrt{k_x^2 + k_y^2}$ of the wave vectors $\mathbf{k}_j$ in media of permeability μ_j and permittivity ϵ_j, with magnitude $k_j = \sqrt{\epsilon_j \mu_j}\omega/c$ and normal component $k_{j,\perp} = \sqrt{k_j^2 - k_\parallel^2}$, and r_α denote the Fresnel reflection coefficients for light with polarization $\alpha \in \{{\rm s}, {\rm p}\}$ impinging on the stratified medium.

Quantum surface corrections contained in the Feibelman d-parameters $d_\perp$ and $d_\parallel$ are incorporated in the optical response of an interface through the modified electromagnetic boundary conditions [12]

$$\hat{\mathbf{n}} \times (\mathbf{E}_2 - \mathbf{E}_1) = -d_\perp \hat{\mathbf{n}} \times [\nabla \hat{\mathbf{n}} \cdot (\mathbf{E}_2 - \mathbf{E}_1)], \tag{S12a}$$

$$\hat{\mathbf{n}} \cdot (\mathbf{D}_2 - \mathbf{D}_1) = d_\parallel \nabla_\parallel [\hat{\mathbf{n}} \times (\mathbf{D}_2 - \mathbf{D}_1) \times \hat{\mathbf{n}}], \tag{S12b}$$

where the subscripts 1 and 2 correspond to regions characterized by different dielectric permittivity and the normal vector $\hat{\mathbf{n}}$ points from medium 1 to 2. In the case of a single metal-dielectric interface where the dielectric and metal permittivity (permeability) are denoted by $\epsilon_{\rm d}$ ($\mu_{\rm d}$) and $\epsilon_{\rm m}$ ($\mu_{\rm m}$), respectively, the surface-corrected Fresnel reflection and transmission coefficients for light impinging on the metal from the dielectric are expressed in terms of the Feibelman d-parameters according to

$$r_{\rm s}^{\rm dm} = \frac{\mu_{\rm m} k_{{\rm d},z} - \mu_{\rm d} k_{{\rm m},z} + {\rm i}(\epsilon_{\rm m} - \epsilon_{\rm d})\mu_{\rm m}\mu_{\rm d} k_0^2 d_\parallel}{\mu_{\rm m} k_{{\rm d},z} + \mu_{\rm d} k_{{\rm m},z} - {\rm i}(\epsilon_{\rm m} - \epsilon_{\rm d})\mu_{\rm m}\mu_{\rm d} k_0^2 d_\parallel}, \tag{S13a}$$

$$t_{\rm s}^{\rm dm} = \frac{2\mu_{\rm m} k_{{\rm d},z}}{\mu_{\rm m} k_{{\rm d},z} + \mu_{\rm d} k_{{\rm m},z} - {\rm i}(\epsilon_{\rm m} - \epsilon_{\rm d})\mu_{\rm m}\mu_{\rm d} k_0^2 d_\parallel}, \tag{S13b}$$

for TE polarized light and

$$r_{\rm p}^{\rm dm} = \frac{\epsilon_{\rm m} k_{{\rm d},z} - \epsilon_{\rm d} k_{{\rm m},z} + {\rm i}(\epsilon_{\rm m} - \epsilon_{\rm d})(k_\parallel^2 d_\perp - k_{{\rm d},z} k_{{\rm m},z} d_\parallel)}{\epsilon_{\rm m} k_{{\rm d},z} + \epsilon_{\rm d} k_{{\rm m},z} - {\rm i}(\epsilon_{\rm m} - \epsilon_{\rm d})(k_\parallel^2 d_\perp + k_{{\rm d},z} k_{{\rm m},z} d_\parallel)}, \tag{S14a}$$

$$t_{\rm p}^{\rm dm} = \frac{2\epsilon_{\rm m} k_{{\rm d},z}}{\epsilon_{\rm m} k_{{\rm d},z} + \epsilon_{\rm d} k_{{\rm m},z} - {\rm i}(\epsilon_{\rm m} - \epsilon_{\rm d})(k_\parallel^2 d_\perp + k_{{\rm d},z} k_{{\rm m},z} d_\parallel)}, \tag{S14b}$$

for TM polarized light [12]. For light impinging from the metal side on the dielectric, the surface-corrected Fresnel coefficients do not obey the usual symmetry relations $r_\alpha^{\rm md} = -r_\alpha^{\rm dm}$, and are instead given by

$$r_{\rm s}^{\rm md} = \frac{\mu_{\rm d} k_{{\rm m},z} - \mu_{\rm m} k_{{\rm d},z} + {\rm i}(\epsilon_{\rm m} - \epsilon_{\rm d})\mu_{\rm m}\mu_{\rm d} k_0^2 d_\parallel}{\mu_{\rm m} k_{{\rm d},z} + \mu_{\rm d} k_{{\rm m},z} - {\rm i}(\epsilon_{\rm m} - \epsilon_{\rm d})\mu_{\rm m}\mu_{\rm d} k_0^2 d_\parallel}, \tag{S15a}$$

$$t_{\rm s}^{\rm md} = \frac{2\mu_{\rm d} k_{{\rm m},z}}{\mu_{\rm m} k_{{\rm d},z} + \mu_{\rm d} k_{{\rm m},z} - {\rm i}(\epsilon_{\rm m} - \epsilon_{\rm d})\mu_{\rm m}\mu_{\rm d} k_0^2 d_\parallel}, \tag{S15b}$$

$$r_{\rm p}^{\rm md} = \frac{\epsilon_{\rm d} k_{{\rm m},z} - \epsilon_{\rm m} k_{{\rm d},z} + {\rm i}(\epsilon_{\rm m} - \epsilon_{\rm d})(k_\parallel^2 d_\perp - k_{{\rm d},z} k_{{\rm m},z} d_\parallel)}{\epsilon_{\rm m} k_{{\rm d},z} + \epsilon_{\rm d} k_{{\rm m},z} - {\rm i}(\epsilon_{\rm m} - \epsilon_{\rm d})(k_\parallel^2 d_\perp + k_{{\rm d},z} k_{{\rm m},z} d_\parallel)}, \tag{S15c}$$

$$t_{\rm p}^{\rm md} = \frac{2\epsilon_{\rm d} k_{{\rm m},z}}{\epsilon_{\rm m} k_{{\rm d},z} + \epsilon_{\rm d} k_{{\rm m},z} - {\rm i}(\epsilon_{\rm m} - \epsilon_{\rm d})(k_\parallel^2 d_\perp + k_{{\rm d},z} k_{{\rm m},z} d_\parallel)}. \tag{S15d}$$

Incidentally, in the nonretarded limit, i.e., taking $c \to \infty$, and assuming nonmagnetic media, $\mu_{\rm d} = \mu_{\rm m} = 1$, the Green's functions in Eq. (S11) associated with a dipole positioned above or in a semi-infinite metal film reduce to the closed-form expressions

$$\mathcal{G}_{\omega,\perp}^{\rm ref,\pm} = -\frac{c^2}{4\pi\epsilon_1\omega^2}\frac{d_\perp + d_\parallel}{d_\perp - d_\parallel}\left\{\left(\eta \pm \frac{1}{d_\perp + d_\parallel}\right)\left[\frac{1}{4z^2} + \frac{\eta}{2z} + \eta^2 {\rm e}^{-2\eta z} E_1(-2\eta z)\right] + \frac{1}{4z^3}\right\} \tag{S16a}$$

for a dipole oriented perpendicular to the film and

$$\mathcal{G}_{\omega,\parallel}^{\rm ref,\pm} = \frac{\zeta}{8\pi}{\rm e}^{-2\zeta z} E_1(-2\zeta z) + \frac{1}{2}\mathcal{G}_{\omega,\perp}^{\rm ref,\pm} \tag{S16b}$$

for a dipole oriented parallel to the film, where $\eta = (\epsilon_{\rm m} + \epsilon_{\rm d})/\left[(\epsilon_{\rm m} - \epsilon_{\rm d})(d_\perp - d_\parallel)\right]$, $\zeta = (\epsilon_{\rm m} - \epsilon_{\rm d})\omega^2 d_\parallel / 2c^2$, E_1 denotes the exponential integral, and the positive (negative) sign corresponds to a dipole situated in the dielectric (metal) medium. The above expressions are analogous to the Green's functions obtained in Ref. [4] for an extended graphene sheet characterized by a two-dimensional conductivity, and generally agree well with the retarded calculation when the dipole is within $\sim 10\,{\rm nm}$ of the interface, as we show in Fig. S2.

S3. OPTICAL RESPONSE OF SPHERICAL GEOMETRIES

In general, the optical response of a spherically-symmetric nanoparticle is characterized by the Green's function describing the electric field produced at $\mathbf{r}$ by a dipole with moment $\mathbf{p}$ located at $\mathbf{r}'$ in a medium with permittivity ϵ_1 according to $\mathbf{E}(\mathbf{r}) = \mu_0\omega^2\mathcal{G}_\varepsilon^{\rm ref}(\mathbf{r},\mathbf{r}')\mathbf{p}$. For an emitter with transition dipole moment oriented radially away from the spherical particle, the Purcell factor and Lamb shift are quantified by the Green's function

$$\mathcal{G}_{\omega,\perp}^{\rm ref} = \frac{1}{\omega^2\mu_0}\sum_l \left(\frac{l+1}{4\pi\epsilon_0\epsilon_1 r^{l+2}}\right)^2 \alpha_l, \tag{S17}$$

where α_l is the multipolar polarizability of the particle. In what follows, we outline derivations of the multipolar polarizability for homogeneous and core-shell spherical nanoparticles in both quasistatic and retarded descriptions.

A. Quasistatic regime

In the quasistatic approximation, we can apply the boundary conditions of Eq. (S12) to a spherical interface where $\hat{\mathbf{n}} = \hat{\mathbf{r}}$ in terms of scalar potentials $\mathbf{E}_j = -\nabla\Phi_j$ as

$$-\hat{\mathbf{r}} \times \nabla(\Phi_2 - \Phi_1) = d_\perp \hat{\mathbf{r}} \times \nabla_\parallel \frac{\partial}{\partial r}(\Phi_2 - \Phi_1), \tag{S18a}$$

$$\frac{\partial}{\partial r}(\epsilon_2\Phi_2 - \epsilon_1\Phi_1) = d_\parallel \nabla_\parallel \cdot [\hat{\mathbf{r}} \times \nabla(\epsilon_2\Phi_2 - \epsilon_1\Phi_1) \times \hat{\mathbf{r}}]. \tag{S18b}$$

1. Homogeneous sphere

For a homogeneous spherical nanoparticle of radius a and permittivity ϵ_2 in a medium with permittivity ϵ_1, we expand the potential satisfying the Laplace equation $\nabla^2\Phi = 0$ in spherical harmonics Y_l^m according to

$$\Phi(r,\theta,\varphi) = \sum_{lm} Y_l^m(\theta,\varphi) \times \begin{cases} A_l\mu^l, & r \le a, \\ \mu^l + B_l\mu^{-l-1}, & r > a, \end{cases} \tag{S19}$$

where $\mu \equiv r/a$. Inserting the above solutions in Eqs. (S12) and simplifying the resulting expressions with the relation

$$\hat{\mathbf{r}} \times \nabla = \hat{\mathbf{r}} \times \nabla_\parallel = -\hat{\theta}\frac{1}{r\sin\theta}\frac{\partial}{\partial\varphi} + \hat{\varphi}\frac{1}{r}\frac{\partial}{\partial\theta} = -\frac{\mathrm{i}}{\hbar a\mu}\mathbf{L}, \tag{S20}$$

where $\mathbf{L}$ denotes the quantum mechanical orbital angular momentum operator satisfying $L^2 Y_l^m = \hbar^2 l(l+1)Y_l^m$, the system of equations governing the coefficients A_l and B_l are isolated using the orthonormality of spherical harmonics and expressed in terms of normalized parameters $\tilde{d}_{\perp,\parallel} \equiv d_{\perp,\parallel}/a$ as

$$1 + l\tilde{d}_\perp - \big(1 + l\tilde{d}_\perp\big)A_l + \big[1 - (l+1)\tilde{d}_\perp\big]B_l = 0, \tag{S21a}$$

$$l\epsilon_1\big[1 + (l+1)\tilde{d}_\parallel\big] - l\epsilon_2\big[1 + (l+1)\tilde{d}_\parallel\big]A_l - \epsilon_1(l+1)\big(1 - l\tilde{d}_\parallel\big)B_l = 0. \tag{S21b}$$

The multipolar polarizability $\alpha_l = -4\pi\epsilon_0\epsilon_1 a^{2l+1}B_l$ is then obtained as

$$\alpha_l = 4\pi\epsilon_0\epsilon_1 a^{2l+1}\frac{l(\epsilon_2 - \epsilon_1)\big[1 + l\tilde{d}_\perp + (l+1)\tilde{d}_\parallel\big]}{l\epsilon_2 + (l+1)\epsilon_1 - l(l+1)(\epsilon_2 - \epsilon_1)(\tilde{d}_\perp - \tilde{d}_\parallel)} \tag{S22}$$

by retaining only linear terms in $\tilde{d}_{\perp,\parallel}$ [12].

2. Core-shell nanoparticle

For a spherical core-shell nanoparticle of inner radius a and outer radius b, the potential is expressed as

$$\Phi(r,\theta,\varphi) = \sum_l Y_l^m(\theta,\varphi) \times \begin{cases} A_l a^{-l} r^l, & r \le a, \\ B_l r^{-1-1} + C_l r^l, & a < r \le b, \\ b^{-l} r^l + D_l b^{l+1} r^{-l-1}, & r > b, \end{cases} \tag{S23}$$

where the core, shell, and exterior permittivity are denoted as ϵ_3, ϵ_2, and ϵ_1, respectively. Following the procedure above for the boundary conditions at $r = a$ and $r = b$, the coefficients are isolated as

$$A_l = \epsilon_1\epsilon_2\Big(\alpha^{(a)}\delta^{(a)} + \beta^{(a)}\gamma^{(a)}\Big)\Big(\alpha^{(b)}\delta^{(b)} + \beta^{(b)}\gamma^{(b)}\Big)\mathcal{F}_l(a/b)^l, \tag{S24a}$$

$$B_l = \alpha^{(a)}\gamma^{(a)}\epsilon_1(\epsilon_3 - \epsilon_2)\Big(\alpha^{(b)}\delta^{(b)} + \beta^{(b)}\gamma^{(b)})\Big)\mathcal{F}_l a^{l+1}(a/b)^l, \tag{S24b}$$

$$C_l = -\epsilon_1\Big(\epsilon_2\alpha^{(a)}\delta^{(a)} + \epsilon_3\beta^{(a)}\gamma^{(a)}\Big)\Big(\alpha^{(b)}\delta^{(b)} + \beta^{(b)}\gamma^{(b)}\Big)\mathcal{F}_l b^{-l}, \tag{S24c}$$

$$D_l = -\Big[\Big(\epsilon_3\beta^{(a)}\gamma^{(a)} + \epsilon_2\alpha^{(a)}\delta^{(a)}\Big)(\epsilon_2 - \epsilon_1)\alpha^{(b)}\gamma^{(b)} + (\epsilon_3 - \epsilon_2)\alpha^{(a)}\gamma^{(a)}\Big(\epsilon_2\alpha^{(b)}\delta^{(b)} + \epsilon_1\beta^{(b)}\gamma^{(b)}\Big)(a/b)^{2l+1}\Big]\mathcal{F}_l, \tag{S24d}$$

where the quantities $\alpha^{(R)} = 1 + l\tilde{d}_\perp^{(R)}$, $\beta^{(R)} = 1 - (l+1)\tilde{d}_\perp^{(R)}$, $\gamma^{(R)} = l\left[1 + (l+1)\tilde{d}_\parallel^{(R)}\right]$, $\delta^{(R)} = (l+1)\left(1 - l\tilde{d}_\parallel^{(R)}\right)$, and

$$\mathcal{F}_l = \left[\left(\epsilon_3\beta^{(a)}\gamma^{(a)} + \epsilon_2\alpha^{(a)}\delta^{(a)}\right)\left(\epsilon_2\beta^{(b)}\gamma^{(b)} + \epsilon_1\alpha^{(b)}\delta^{(b)}\right) + (\epsilon_3 - \epsilon_2)(\epsilon_2 - \epsilon_1)\alpha^{(a)}\beta^{(b)}\gamma^{(a)}\delta^{(b)}(a/b)^{2l+1}\right]^{-1} \tag{S25}$$

are introduced to simplify the notation, and expressed in terms of normalized parameters $\tilde{d}_{\perp,\parallel}^{(R)} = d_{\perp,\parallel}^{(R)}/R$ for $R \in \{a, b\}$ with $d_{\perp,\parallel}^{(R)}$ indicating the d-parameters corresponding to the interface at $r = R$. The multipolar polarizability of the core-shell particle is then $\alpha_l = -4\pi\epsilon_0\epsilon_1 b^{2l+1} D_l$.

B. Mie Theory

Relaxing the quasistatic approximation used in the previous subsection, the surface-corrected optical response of a spherical nanoparticle is described by incorporating Feibelman d-parameters in Mie theory. Here we present a derivation based on the formalism of Ref. [13] that recovers the result obtained for a homogeneous spherical nanoparticle first reported in Ref. [12]. We then apply the same procedure to describe the fully retarded response of a spherical core-shell nanoparticle.

The electric field in region j satisfying the wave equation $\nabla^2\mathbf{E}(\mathbf{r},\omega) + k_j^2\mathbf{E}_j(\mathbf{r},\omega) = 0$ is expressed as a sum of incoming and outgoing fields $\mathbf{E}_j = \mathbf{E}_j^{\rm in} + \mathbf{E}_j^{\rm out}$ given by

$$\mathbf{E}_j^{\rm in}(\mathbf{r},\omega) = \sum_{lm} E_l\left[b_{j,lm}^{\rm in} g_{j,l}(k_j r)\mathbf{X}_{lm}(\theta,\varphi) + \frac{\mathrm{i}}{k_j} a_{j,lm}^{\rm in}\nabla\times f_{j,l}(k_j r)\mathbf{X}_{lm}(\theta,\varphi)\right], \tag{S26a}$$

$$\mathbf{E}_j^{\rm out}(\mathbf{r},\omega) = -\sum_{lm} E_l\left[b_{j,lm}^{\rm out} q_{j,l}(k_j r)\mathbf{X}_{lm}(\theta,\varphi) + \frac{\mathrm{i}}{k_j} a_{j,lm}^{\rm out}\nabla\times p_{j,l}(k_j r)\mathbf{X}_{lm}(\theta,\varphi)\right], \tag{S26b}$$

where the functions containing the radial dependence are chosen as spherical Bessel functions j_l or Hankel functions of the first kind $h_l^{(1)}$ according to geometrical considerations, while the vector spherical harmonics are defined in terms of the angular momentum operator $\mathbf{L}$ as

$$\mathbf{X}_{lm}(\theta,\varphi) = \frac{1}{\sqrt{l(l+1)}}\mathbf{L}Y_l^m(\theta,\varphi) \tag{S27}$$

and satisfy the orthonormality relations [13]

$$\oint d\Omega\mathbf{X}_{l'm'}^*(\theta,\varphi)\cdot[f_l(r)\mathbf{X}_{lm}(\theta,\varphi)] = f_l(r)\delta_{l'l}\delta_{m'm}, \tag{S28a}$$

$$\oint d\Omega\mathbf{X}_{l'm'}^*(\theta,\varphi)\cdot[\nabla\times f_l(r)\mathbf{X}_{lm}(\theta,\varphi)] = 0, \tag{S28b}$$

$$\oint d\Omega\mathbf{r}\times\mathbf{X}_{l'm'}^*(\theta,\varphi)\cdot[f_l(r)\mathbf{X}_{lm}(\theta,\varphi)] = 0, \tag{S28c}$$

$$\oint d\Omega\mathbf{r}\times\mathbf{X}_{l'm'}^*(\theta,\varphi)\cdot[\nabla\times f_l(r)\mathbf{X}_{lm}(\theta,\varphi)] = \left[\frac{d}{dr}rf_l(r)\right]\delta_{l'l}\delta_{m'm}. \tag{S28d}$$

where $\Omega \equiv (\theta,\varphi)$ denote angular coordinates and $f_l(r)$ are linear combinations of spherical Bessel functions and Hankel functions of the first kind. Analogous expressions for the magnetic field $\mathbf{H}_j = \mathbf{H}_j^{\rm in} + \mathbf{H}_j^{\rm out}$ satisfying the wave equation $\nabla^2\mathbf{H}_j + k_j^2\mathbf{H} = 0$ are obtained as

$$\mathbf{H}_j^{\rm in}(\mathbf{r},\omega) = \frac{1}{Z_j}\sum_{lm} E_l\left[a_{j,lm}^{\rm in} f_{j,l}(k_j r)\mathbf{X}_{lm}(\theta,\varphi) - \frac{\mathrm{i}}{k_j} b_{j,lm}^{\rm in}\nabla\times g_{j,l}(k_j r)\mathbf{X}_{lm}(\theta,\varphi)\right], \tag{S29a}$$

$$\mathbf{H}_j^{\rm out}(\mathbf{r},\omega) = -\frac{1}{Z_j}\sum_{lm} E_l\left[a_{j,lm}^{\rm out} p_{j,l}(k_j r)\mathbf{X}_{lm}(\theta,\varphi) - \frac{\mathrm{i}}{k_j} b_{j,lm}^{\rm out}\nabla\times q_{j,l}(k_j r)\mathbf{X}_{lm}(\theta,\varphi)\right]. \tag{S29b}$$

For a spherical interface at $r = R$ with normal vector pointing outwards from medium 1 to medium 2, the surface-corrected electromagnetic boundary conditions are written as [12]

$$\left(\mathbf{E}_{2,\Omega}^{\rm in} + \mathbf{E}_{2,\Omega}^{\rm out} - \mathbf{E}_{1,\Omega}^{\rm in} - \mathbf{E}_{1,\Omega}^{\rm out}\right)\Big|_{r=R} = -d_\perp\,\nabla_\Omega\left(E_{2,r}^{\rm in} + E_{2,r}^{\rm out} - E_{1,r}^{\rm in} - E_{1,r}^{\rm out}\right)\Big|_{r=R}, \tag{S30a}$$

$$\left(\mathbf{H}_{2,\Omega}^{\rm in} + \mathbf{H}_{2,\Omega}^{\rm out} - \mathbf{H}_{1,\Omega}^{\rm in} - \mathbf{H}_{1,\Omega}^{\rm out}\right)\Big|_{r=R} = \mathrm{i}\omega d_\parallel\,\left(\mathbf{D}_{2,\Omega}^{\rm in} + \mathbf{D}_{2,\Omega}^{\rm out} - \mathbf{D}_{1,\Omega}^{\rm in} - \mathbf{D}_{1,\Omega}^{\rm out}\right)\times\hat{\mathbf{n}}\Big|_{r=R}. \tag{S30b}$$

Making use of the orthonormality relations in Eqs. (S28), we isolate a system of equations that relates the coefficients a_{lm} and b_{lm} in the incoming and outgoing regions as:

$$b^{\rm in}_{2,lm}g_{2,l}(x_2)-b^{\rm out}_{2,lm}q_{2,l}(x_2)=b^{\rm in}_{1,lm}g_{1,l}(x_1)-b^{\rm out}_{1,lm}q_{1,l}(x_1), \tag{S31a}$$

$$\frac{1}{k_2}\left\{a^{\rm in}_{2,lm}\left[F'_{2,l}(x_2)+\bar{d}_\perp f_{2,l}(x_2)\right]-a^{\rm out}_{2,lm}\left[P'_{2,l}(x_2)+\bar{d}_\perp p_{2,l}(x_2)\right]\right\}$$
$$=\frac{1}{k_1}\left\{a^{\rm in}_{1,lm}\left[F'_{1,l}(x_1)+\bar{d}_\perp f_{1,l}(x_1)\right]-a^{\rm out}_{1,lm}\left[P'_{1,l}(x_1)+\bar{d}_\perp p_{1,l}(x_1)\right]\right\}, \tag{S31b}$$

$$\frac{1}{Z_2}\left\{a^{\rm in}_{2,lm}\left[f_{2,l}(x_2)+\bar{d}_\parallel F'_{2,l}(x_2)\right]-a^{\rm out}_{2,lm}\left[p_{2,l}(x_2)+\bar{d}_\parallel P'_{2,l}(x_2)\right]\right\}$$
$$=\frac{1}{Z_1}\left\{a^{\rm in}_{1,lm}\left[f_{1,l}(x_1)+\bar{d}_\parallel F'_{1,l}(x_1)\right]-a^{\rm out}_{1,lm}\left[p_{1,l}(x_1)+\bar{d}_\parallel P'_{1,l}(x_1)\right]\right\}, \tag{S31c}$$

$$\frac{1}{k_2 Z_2}\left\{b^{\rm in}_{2,lm}\left[\bar{d}_\parallel x_2^2 g_{2,l}(x_2)-G'_{2,l}(x_2)\right]+b^{\rm out}_{2,lm}\left[Q'_{2,l}(x_2)-\bar{d}_\parallel x_2^2 q_{2,l}(x_2)\right]\right\}$$
$$=\frac{1}{k_1 Z_1}\left\{b^{\rm in}_{1,lm}\left[\bar{d}_\parallel x_1^2 g_{1,l}(x_1)-G'_{1,l}(x_1)\right]+b^{\rm out}_{1,lm}\left[Q'_{1,l}(x_1)-\bar{d}_\parallel x_1^2 q_{1,l}(x_1)\right]\right\}, \tag{S31d}$$

where the quantities $F_{j,l}(x)\equiv xf_{j,l}(x)$, $G_{j,l}(x)\equiv xg_{j,l}(x)$, $P_{j,l}(x)\equiv xp_{j,l}(x)$, and $Q_{j,l}(x)\equiv xq_{j,l}(x)$, along with their derivatives indicated by prime marks, are evaluated at $x_j\equiv k_jR$, while $\bar{d}_\perp\equiv l(l+1)d_\perp/R$ and $\bar{d}_\parallel\equiv d_\parallel/R$ are normalized d-parameters.

1. *Homogeneous sphere*

The optical response of a spherical particle with permittivity ϵ_2 and radius a embedded in a homogeneous medium with permittivity ϵ_1 is described by the fields

$$\mathbf{E}=\begin{cases}\mathbf{E}_2^{\rm sca}, & r\le a,\\ \mathbf{E}_1^{\rm inc}+\mathbf{E}_1^{\rm sca}, & r>a,\end{cases} \tag{S32}$$

where $\mathbf{E}_j^{\rm inc}$ and $\mathbf{E}_j^{\rm sca}$ denote the incoming and scattered fields, respectively, in region j with permittivity ϵ_j. To satisfy the boundary conditions in the limits $r\to 0$ and $r\to\infty$, the radial components of the scattered fields $\mathbf{E}_2^{\rm sca}$ and $\mathbf{E}_1^{\rm sca}$ are chosen as spherical Bessel and Hankel functions, respectively, and the coefficients of (S26) are obtained at linear order in the d-parameters as

$$\frac{a^{\rm out}_{2,lm}}{a^{\rm in}_{1,lm}}=\frac{\sqrt{\epsilon_1\epsilon_2}\left[f_{1,l}(x_1)\xi'_l(x_1)-h_l^{(1)}(x_1)F'_{1,l}(x_1)\right]}{\epsilon_1 h^{(1)}(x_1)\Psi'_l(x_2)-\epsilon_2 j_l(x_2)\xi'_l(x_1)+(\epsilon_1-\epsilon_2)\left[\bar{d}_\perp h_l^{(1)}(x_1)j_l(x_2)+\bar{d}_\parallel\psi'_l(x_2)\xi'_l(x_1)\right]}, \tag{S33a}$$

$$\frac{b^{\rm out}_{2,lm}}{b^{\rm in}_{1,lm}}=\frac{G'_{1,l}(x_1)h^{(1)}(x_1)-g_{1,l}(x_1)\xi'_l(x_1)}{j_l(x_2)\xi'_l(x_1)-h^{(1)}(x_1)\psi'_l(x_2)-\bar{d}_\parallel h^{(1)}(x_1)j_l(x_2)(x_1^2-x_2^2)}, \tag{S33b}$$

$$\frac{a^{\rm out}_{1,lm}}{a^{\rm in}_{1,lm}}=\frac{\epsilon_2F'_{1,l}(x_1)j_l(x_2)-\epsilon_1 f_{1,l}(x_1)\psi'_l(x_2)+(\epsilon_2-\epsilon_1)(\bar{d}_\perp f_{1,l}(x_1)j_l(x_2)+\bar{d}_\parallel F'_{1,l}(x_1)\psi'_l(x_2))}{\epsilon_2 j_l(x_2)\xi'_l(x_1)-\epsilon_1 h^{(1)}(x_1)\psi'_l(x_2)+(\epsilon_2-\epsilon_1)(\bar{d}_\perp h^{(1)}(x_1)j_l(x_2)+\bar{d}_\parallel\psi'_l(x_2)\xi'_l(x_1))}, \tag{S33c}$$

$$\frac{b^{\rm out}_{1,lm}}{b^{\rm in}_{1,lm}}=\frac{g_{1,l}(x_1)\psi'_l(x_2)-G'_{1,l}(x_1)j_l(x_2)-\bar{d}_\parallel g_{1,l}(x_1)j_l(x_2)\left(x_2^2-x_1^2\right)}{h^{(1)}(x_1)\psi'_l(x_2)-j_l(x_2)\xi'_l(x_1)-\bar{d}_\parallel j_l(x_2)h^{(1)}(x_1)(x_2^2-x_1^2)}. \tag{S33d}$$

To determine the Green's function describing the field at the location of a dipole produced by its reflection in the spherical nanoparticle, the scattered field is expanded as

$$\mathbf{E}_1^{\rm sca}=-\sum_{lm}E_l\left\{b^{\rm out}_{1,lm}h_l^{(1)}(x_1)\mathbf{X}_{lm}(\theta,\varphi)+\right.$$
$$\left.+\frac{a^{\rm out}_{1,lm}}{k_1r^2}\left[{\rm i}\xi'_l(x_1)\mathbf{r}\times\mathbf{X}_{lm}(\theta,\varphi)-\sqrt{l(l+1)}h_l^{(1)}(x_1)\mathbf{r}Y_l^m(\theta,\varphi)\right]\right\}. \tag{S34}$$

Without loss of generality, the response of a dipole oriented normally to the spherical particle at a distance r from its center is found by projecting $\hat{\mathbf{z}}\cdot\mathbf{E}_1^{\rm sca}=p\omega^2\mu_0\hat{\mathbf{z}}\cdot\mathcal{G}_\omega^{\rm ref}\cdot\hat{\mathbf{z}}$ to isolate

$$\mathcal{G}^{\rm ref}_{\omega,\perp}(r)=-\frac{{\rm i}k_1}{4\pi}\sum_l l(l+1)(2l+1)\frac{a^{\rm out}_{1,lm}}{a^{\rm in}_{1,lm}}\left[\frac{h_l^{(1)}(k_1r)}{k_1r}\right]^2. \tag{S35}$$

In a similar fashion, we obtain the reflected Green's function corresponding to a dipole $\mathbf{p}_\pm=p(\hat{\mathbf{x}}\pm{\rm i}\hat{\mathbf{y}})$ as

$$\mathcal{G}^{\rm ref}_{\omega,\pm}(r)=-\frac{{\rm i}k_1}{8\pi}\sum_l(2l+1)\left\{\frac{a^{\rm out}_{1,lm}}{a^{\rm in}_{1,lm}}\left(\frac{\xi_l'(x_1)}{x_1}\right)^2+\frac{b^{\rm out}_{1,lm}}{b^{\rm in}_{1,lm}}\left[h_l^{(1)}(x_1)\right]^2\right\}. \tag{S36}$$

To quantify the total scattered field produced by a dipole located at $\mathbf{r}$ near a spherical nanoparticle, we compute the total Green's function at a detection position $\mathbf{r}_{\rm D}$ as

$$\mathcal{G}_{\omega,\perp}(\mathbf{r}_{\rm D},\mathbf{r})=\mathcal{G}^{(0)}_{\omega,\perp}-\frac{{\rm i}k_1}{4\pi}\sum_l l(l+1)(2l+1)\frac{a^{\rm out}_{1,lm}}{a^{\rm in}_{1,lm}}\frac{h_l^{(1)}(k_1r)}{k_1r}\frac{h_l^{(1)}(k_1r_{\rm D})}{k_1r_{\rm D}}, \tag{S37}$$

for a dipole oriented normally to the spherical particle, where the bare Green's dyadic is given by

$$\mathcal{G}^{(0)}_\omega(\mathbf{r},\mathbf{r}')=\frac{{\rm e}^{{\rm i}kR}}{4\pi R}\left[\left(1+\frac{{\rm i}kR-1}{k^2R^2}\right)\mathbb{1}+\frac{3-3{\rm i}kR-k^2R^2}{k^2R^4}\mathbf{R}\otimes\mathbf{R}\right] \tag{S38}$$

and expressed in terms of $\mathbf{R}=\mathbf{r}-\mathbf{r}'$ and the wave vector k of an arbitrary homogeneous medium, while

$$\mathcal{G}_{\omega,\pm}(\mathbf{r}_{\rm D},\mathbf{r})=\mathcal{G}^{(0)}_{\omega,\pm}(\mathbf{r}_{\rm D},\mathbf{r})-\frac{{\rm i}k_1}{8\pi}\sum_l(2l+1)\left[\frac{a^{\rm out}_{1,lm}}{a^{\rm in}_{1,lm}}\frac{\xi_l'(k_1r)}{k_1r}\frac{\xi_l'(k_1r_{\rm D})}{k_1r_{\rm D}}+\frac{b^{\rm out}_{1,lm}}{b^{\rm in}_{1,lm}}h_l^{(1)}(k_1r)h_l^{(1)}(k_1r_{\rm D})\right] \tag{S39}$$

for a dipole oriented tangentially to the spherical particle.

2. *Core-shell nanoparticle*

The above treatment of a homogeneous particle is straightforwardly extended to the case of a spherical core-shell nanoparticle by considering an additional interface, such that the electric field is given as

$$\mathbf{E}=\begin{cases}\mathbf{E}_3^{\rm sca}, & r\le a,\\ \mathbf{E}_2^{\rm inc}+\mathbf{E}_2^{\rm sca}, & a<r\le b,\\ \mathbf{E}_1^{\rm inc}+\mathbf{E}_1^{\rm sca}, & r>b,\end{cases} \tag{S40}$$

where ϵ_1 is the permittivity of the surrounding environment, ϵ_2 is the permittivity of the shell with inner radius a and outer radius b, and ϵ_3 is the permittivity of the core with radius a. Choosing the appropriate spherical Bessel and Hankel functions in Eqs. (S26) and projecting on vector spherical harmonics leads to the Mie coefficients

$$b^{\rm out}_{2,lm}h_l^{(1)}(k_2a)-b^{\rm in}_{2,lm}j_l(k_2a)=b^{\rm out}_{3,lm}j_l(k_3a), \tag{S41a}$$

$$a^{\rm out}_{2,lm}\left[\xi_l'(k_2a)+\bar{d}_\perp^{3,2}h_l^{(1)}(k_2a)\right]-a^{\rm in}_{2,lm}\left[\psi_l'(k_2a)+\bar{d}_\perp^{3,2}j_l(k_2a)\right]=\sqrt{\frac{\epsilon_2}{\epsilon_3}}a^{\rm out}_{3,lm}\left[\psi_l'(k_3a)+j_l(k_3a)\bar{d}_\perp^{3,2}\right], \tag{S41b}$$

$$a^{\rm out}_{2,lm}\left[h_l^{(1)}(k_2a)+\bar{d}_\parallel^{3,2}\xi_l'(k_2a)\right]-a^{\rm in}_{2,lm}\left[j_l(k_2a)+\bar{d}_\parallel^{3,2}\psi_l'(k_2a)\right]=\sqrt{\frac{\epsilon_3}{\epsilon_2}}a^{\rm out}_{3,lm}\left[j_l(k_3a)+\bar{d}_\parallel^{3,2}\psi_l'(k_3a)\right], \tag{S41c}$$

$$\begin{aligned}b^{\rm out}_{2,lm}\left[\xi_l'(k_2a)-\bar{d}_\parallel^{3,2}(k_2a)^2h_l^{(1)}(k_2a)\right]-b^{\rm in}_{2,lm}\left[\psi_l'(k_2a)-\bar{d}_\parallel^{3,2}(k_2a)^2j_l(k_2a)\right]\\=b^{\rm out}_{3,lm}\left[\psi_l'(k_3a)-\bar{d}_\parallel^{3,2}(k_3a)^2j_l(k_3a)\right],\end{aligned} \tag{S41d}$$

$$b^{\mathrm{out}}_{1,lm} h^{(1)}_l(k_1 b) - b^{\mathrm{in}}_{1,lm} g_{1,l} = b^{\mathrm{out}}_{2,lm} h^{(1)}_l(k_2 b) - b^{\mathrm{in}}_{2,lm} j_l(k_2 b), \tag{S41e}$$

$$a^{\mathrm{out}}_{1,lm}\Big[\xi'_l(k_1 b) + \bar{d}^{2,1}_{\perp} h^{(1)}_l(k_1 b)\Big] - a^{\mathrm{in}}_{1,lm}\Big[F'_{1,l}(k_1 b) + \bar{d}^{2,1}_{\perp} f_{1,l}(k_1 b)\Big] \\ = \frac{\sqrt{\epsilon_1}}{\sqrt{\epsilon_2}}\Big\{a^{\mathrm{out}}_{2,lm}\Big[\xi'_l(k_2 b) + h^{(1)}_l(k_2 b)\bar{d}^{2,1}_{\perp}\Big] - a^{\mathrm{in}}_{2,lm}\Big[\psi'_l(k_2 b) + \bar{d}^{2,1}_{\perp} j_l(k_2 b)\Big]\Big\}, \tag{S41f}$$

$$a^{\mathrm{out}}_{1,lm}\Big[h^{(1)}_l(k_1 b) + \bar{d}^{2,1}_{\parallel}\xi'_l(k_1 b)\Big] - a^{\mathrm{in}}_{1,lm}\Big[f_{1,l}(k_1 b) + \bar{d}^{2,1}_{\parallel} F'_{1,l}(k_1 b)\Big] \\ = \frac{\sqrt{\epsilon_2}}{\sqrt{\epsilon_1}}\Big\{a^{\mathrm{out}}_{2,lm}\Big[h^{(1)}_l(k_2 b) + \bar{d}^{2,1}_{\parallel}\xi'_l(k_2 b)\Big] - a^{\mathrm{in}}_{2,lm}\Big[j_l(k_2 b) + \bar{d}^{2,1}_{\parallel}\psi'_l(k_2 b)\Big]\Big\}, \tag{S41g}$$

$$b^{\mathrm{out}}_{1,lm}\Big[\xi'_l(k_1 b) - \bar{d}^{2,1}_{\parallel}(k_1 b)^2 h^{(1)}_l(k_1 b)\Big] - b^{\mathrm{in}}_{1,lm}\Big[G'_{1,l}(k_1 b) - \bar{d}^{2,1}_{\parallel}(k_1 b)^2 g_{1,l}(k_1 b)\Big] \\ = b^{\mathrm{out}}_{2,lm}\Big[\xi'_l(k_2 b) - \bar{d}^{2,1}_{\parallel}(k_2 b)^2 h^{(1)}_l(k_2 b)\Big] - b^{\mathrm{in}}_{2,lm}\Big[\psi'_l(k_2 b) - \bar{d}^{2,1}_{\parallel}(k_2 b)^2 j_l(k_2 b)\Big]. \tag{S41h}$$

In practice, we use computational symbolic solvers to obtain analytical expressions for the Mie coefficients, which are however too lengthy to be displayed. The Green's functions are calculated in an analogous manner as for the homogeneous spherical nanoparticle.

S4. VALIDITY OF THE QUASISTATIC APPROXIMATION

The role of surface effects in the quantum electrodynamic response are explored in the main text through the Purcell factor and Lamb shift computed in the full electromagnetic calculations that include retardation effects. However, it is instructive to assess the regimes in which the quasistatic approximation can be safely invoked, which, in the case of a single metal-dielectric interface, along with the homogeneous and core-shell spherical particles, leads to analytical expressions for the Green's functions. In Fig. S2 we present the Purcell factor and Lamb shift for emitters in the considered geometries as predicted in both quasistatic (dashed curves) and retarded (solid curves) calculations. The results indicate that the quasistatic approximation is well-justified when treating a dipole $\lesssim 10\,\mathrm{nm}$ from a planar interface or a spherical nanoparticle with outer radius $\lesssim 25\,\mathrm{nm}$.

S5. COMPARISON OF SURFACE RESPONSE CORRECTIONS TO HYDRODYNAMIC SIMULATIONS

The Feibelman d-parameters constitute surface response functions that introduce intuitive leading-order nonlocal corrections to the classical electrodynamic boundary conditions at metal-dielectric interfaces. Our analysis of nonlocal effects in the optical response of a point dipole emitter near metal surfaces is based on d-parameters obtained in the specular-reflection model (SRM) combined with a longitudinal dielectric function based on the hydrodynamic model (HDM). While this approach facilitates analytical descriptions of the nonlocal electrodynamic response at metal-dielectric interfaces in spherical and planar morphologies, the surface and bulk nonlocal response in such systems can be treated by solving the SRM with HDM directly as described in Refs. [14–16]. More specifically, in the quasistatic regime, the reflection coefficient of a planar semi-infinite metal film is given by

$$r^{\mathrm{SRM}}_{\mathrm{p}}(k_\parallel,\omega) = \frac{\epsilon^{\mathrm{SRM}}(k_\parallel,\omega) - \epsilon_{\mathrm{d}}}{\epsilon^{\mathrm{SRM}}(k_\parallel,\omega) + \epsilon_{\mathrm{d}}}, \tag{S42}$$

where ϵ^{SRM} denotes the effective bulk dielectric function of the metal in the SRM, and is expressed in terms of the in-plane wave vector $k_\parallel$ and longitudinal dielectric function ϵ_{L} as [14, 17]

$$\left[\epsilon^{\mathrm{SRM}}(k_\parallel,\omega)\right]^{-1} = \frac{2k_\parallel}{\pi}\int_0^\infty \frac{dk_\perp}{k_\parallel^2 + k_\perp^2}\left[\epsilon_{\mathrm{L}}\Big(\sqrt{k_\parallel^2 + k_\perp^2},\omega\Big)\right]^{-1}. \tag{S43}$$

Invoking the HDM, we write

$$\epsilon_{\mathrm{L}}(k,\omega) = \epsilon_{\mathrm{b}}(\omega) - \frac{\omega_{\mathrm{p}}^2}{\omega^2 + \mathrm{i}\omega\gamma - \beta^2 k^2} \tag{S44}$$

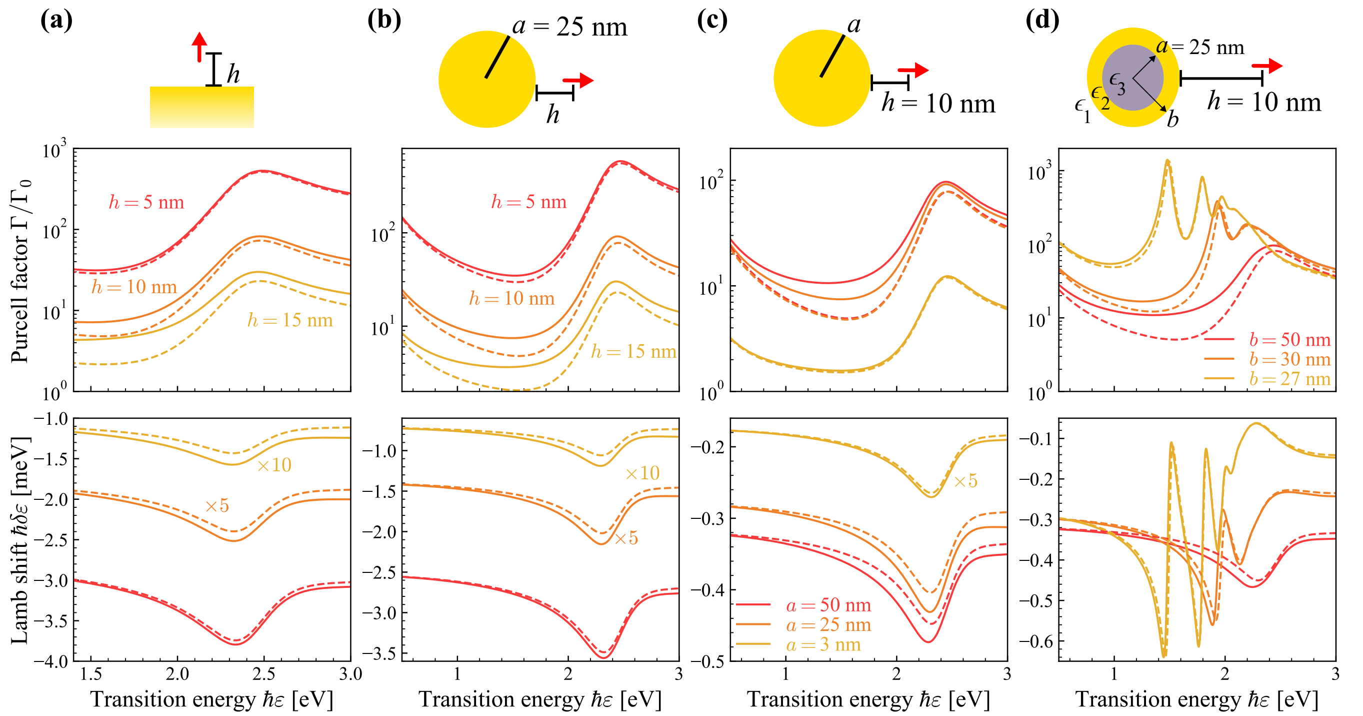

FIG. S2. **Comparing retarded and quasistatic descriptions of the Purcell factor and Lamb shift.** The Purcell factor (upper panels) and Lamb shift (lower panels) predicted for a dipole in air oriented normally to and (a) at a varying distance h above a single gold interface; (b) at a varying distance h from a spherical gold nanoparticle of radius $a = 25\,$nm; (c) at a fixed distance $h = 10\,$nm from a spherical gold particle with varying radius a; (d) at a fixed distance $h = 10\,$nm above a spherical nanoparticle with silica core ($\epsilon = 2.13$) of radius $a = 25\,$nm and gold shell of varying outer radius b. In all cases the transition dipole moment of the emitter is $1\,e{\cdot}$nm and the permittivity of gold is given by the model of Ref. [9].

to evaluate the integral in Eq. (S43) and obtain

$$\epsilon^{\rm SRM}(k_\parallel,\omega) = \frac{\epsilon_{\rm L}(0,\omega)}{1-\omega_{\rm p}^2\beta k_\parallel/\left\{\epsilon_{\rm b}(\omega)(\omega^2+{\rm i}\gamma\omega)\sqrt{\left[\omega_{\rm p}^2/\epsilon_{\rm b}(\omega)\right]-\omega^2-{\rm i}\gamma\omega+\beta^2k_\parallel^2}\right\}}. \tag{S45}$$

For a spherical metal nanoparticle with radius a, the SRM combined with a nonlocal dielectric function $\epsilon(q,\omega)$ leads to the polarizability

$$\alpha_l = 4\pi\epsilon_0\epsilon_{\rm d}a^{2l+1}\frac{1-\epsilon_{\rm d}\frac{2a(2l+1)}{\pi}\int_0^\infty\frac{dq}{\epsilon(q,\omega)}j_l^2(qa)}{1+\frac{l+1}{l}\epsilon_{\rm d}\frac{2a(2l+1)}{\pi}\int_0^\infty\frac{dq}{\epsilon(q,\omega)}j_l^2(qa)}, \tag{S46}$$

as reported in Refs. [15, 16].

In Fig. S3, we compare results reported in the main text for semi-infinite metal films and spherical metal nanoparticles using analytical expressions based on Feibelman d-parameters with those obtained from numerically integrating the full nonlocal problem within the combined SRM and HDM. In particular, we show the Purcell factor for an emitter placed in air at different distances from a planar gold surface (Fig. S3a) and near a spherical gold nanoparticle (Fig. S3b) when nonlocal effects are neglected (dashed curves), approximated using d-parameters (solid curves), and fully included using numerical integration techniques (dot-dashed curves). For the metal film, the Purcell factors obtained in the d-parameter formalism are in excellent agreement with their counterparts computed in SRM+HDM for all considered separation distances, indicating that the d-parameters capture the dominant nonlocal effects in plasmon-emitter interactions for planar geometries. Similar agreement is obtained for spherical metal nanoparticles for the considered particle radii, except for a deviation at lower energies in the case of the smallest NP with radius $a = 3\,$nm. This discrepancy presumably arises due to the fact that the d-parameter correction is based on the response of a planar interface, and thus does not account for additional damping due to nonlocal effects associated with the curvature of the particle on extreme few-nanometer length scales. In principle, such curvature effects could be captured in the Feibelman surface response formalism by introducing wave-vector-dependent d-parameters as proposed in Ref. [18].

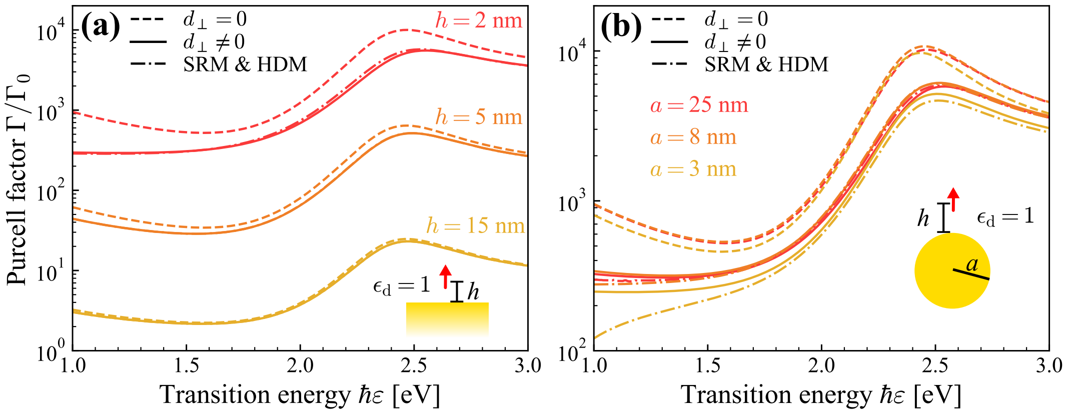

FIG. S3. **Comparison of the Purcell factor predicted using d-parameters with direct calculations of the nonlocal metal response described in the SRM using HDM.** The Purcell factor is computed for the configurations and parameters considered in (a) Fig. 1(c) for a semi-infinite gold film and (b) Fig. 2(b) for a spherical gold nanoparticle when nonlocal effects are omitted (dashed curves), approximated using d-parameters (solid curves), or included directly in the combined SRM and HDM according to Eqs. (S42) and (S46) (dash-dotted curves).

[1] R.-C. Ge, C. Van Vlack, P. Yao, J. F. Young, and S. Hughes, Phys. Rev. B **87**, 205425 (2013).
[2] E. Forati, G. W. Hanson, and S. Hughes, Phys. Rev. B **90**, 085414 (2014).
[3] T. V. C. Antão and N. M. R. Peres, Int. J. Mod. Phys. B **35**, 2130007 (2021).
[4] M. H. Eriksen, J. E. Olsen, C. Wolff, and J. D. Cox, Phys. Rev. Lett. **129**, 253602 (2022).
[5] S. Scheel and S. Y. Buhmann, Acta Phys. Slovaca **58**, 675 (2008).
[6] L. Novotny and B. Hecht, *Principles of nano-optics* (Cambridge University Press, Cambridge, 2012).
[7] P. Yao, C. Van Vlack, A. Reza, M. Patterson, M. Dignam, and S. Hughes, Phys. Rev. B **80**, 195106 (2009).
[8] D. Dzsotjan, J. Kästel, and M. Fleischhauer, Phys. Rev. B **84**, 075419 (2011).
[9] P. G. Etchegoin, E. C. Le Ru, and M. Meyer, J. Chem. Phys. **125**, 164705 (2006).
[10] C. V. Vlack, P. T. Kristensen, and S. Hughes, Phys. Rev. B **85**, 075303 (2012).
[11] P. Meystre and M. Sargent, *Elements of quantum optics* (Springer Science & Business Media, Berlin, 2007).
[12] P. A. D. Gonçalves, T. Christensen, N. Rivera, A.-P. Jauho, N. A. Mortensen, and M. Soljačić, Nat. Commun. **11**, 366 (2020).
[13] U. Hohenester, *Nano and Quantum Optics* (Springer, Cham, 2020).
[14] G. W. Ford and W. H. Weber, Phys. Rep. **113**, 195 (1984).
[15] F. J. García de Abajo, J. Phys. Chem. C **112**, 17983 (2008).
[16] C. David and F. J. García de Abajo, J. Phys. Chem. C **115**, 19470 (2011).
[17] P. A. D. Gonçalves, *Plasmonics and Light–Matter Interactions in Two-Dimensional Materials and in Metal Nanostructures: Classical and Quantum Considerations* (Springer Nature, Cham, 2020).
[18] A. Babaze, E. Ogando, P. E. Stamatopoulou, C. Tserkezis, N. A. Mortensen, J. Aizpurua, A. G. Borisov, and R. Esteban, Opt. Express **30**, 21159 (2022).
